# What, in Fact, Is the Evidence That Vaccinating Healthcare Workers against Seasonal Influenza Protects Their Patients? A Critical Review

**DOI:** 10.1155/2012/205464

**Published:** 2012-11-11

**Authors:** Zvi Howard Abramson

**Affiliations:** ^1^Department of Family Medicine, Hebrew University-Hadassah School of Public Health, Ein Kerem, Jerusalem 91120, Israel; ^2^Beit Hakerem Community Health Center, Clalit Health Services, Haarazim 2, Jerusalem 96182, Israel

## Abstract

*Background and Methods*. Vaccination of all healthcare workers is widely recommended by health authorities and medical institutions and support for mandatory vaccination is increasing. This paper presents the relevant literature and examines the evidence for patient benefit from healthcare worker vaccination. Articles identified by Medline searches and citation lists were inspected for internal and external validity. Emphasis was put on RCTs. The literature on self-protection from vaccination is also presented. *Results*. Published research shows that personal benefit from vaccinating healthy nonelderly adults is small and there is no evidence that it is any different for HCWs. The studies aiming to prove the widespread belief that healthcare worker vaccination decreases patient morbidity and mortality are heavily flawed and the recommendations for vaccination biased. No reliable published evidence shows that healthcare workers' vaccination has substantial benefit for their patients—not in reducing patient morbidity or mortality and not even in increasing patient vaccination rates. *Conclusion*. The arguments for uniform healthcare worker influenza vaccination are not supported by existing literature. The decision whether to get vaccinated should, except possibly in extreme situations, be that of the individual healthcare worker, without legal, institutional, or peer coercion.

## 1. Introduction

Vaccination of all healthcare workers (HCWs) is widely recommended by health authorities and medical institutions [1–3]. This recommendation is based on the argument that because of their proximity to patients, HCW vaccination protects themselves and their patients from influenza.

The growing pressure on HCWs to vaccinate as part of their ethical professional responsibility is illustrated by the statement by the Canadian National Advisory Committee on Immunization for the 2010-2011 season that “in the absence of contraindications, refusal of HCWs who have direct patient contact to be immunized against influenza implies failure in their duty of care to patients” [2]. The fact that this is being taken even a step further with recommendations and pressure on institutions to mandate such vaccination at the expense of individual freedom and as a condition for continued employment [4] increases the urgency of examining the evidence. Is it sufficient for such draconian measures?

The argument in favor of vaccinating all HCWs is based primarily on their obligation to protect their patients. However, in order to give a complete picture, the evidence base concerning personal HCW benefit from vaccination is also presented.

As demonstrated in a study of an educational and promotional intervention in primary care, HCWs' vaccination rates can be increased by an easy intervention [5]. However, does the evidence justify widespread implementation of such programs?

The specific questions regarding benefit from HCW vaccination examined in this paper are detailed in Section 2.

## 2. Methods

The possible reasons for vaccinating HCWs are presented and discussed in turn.

**Proposed Reasons for Vaccinating Healthcare Workers**

- Self-protection
  - All adults (HCWs included) should be vaccinated against influenza.
  - HCWs are at increased risk of infection (and thus also of secondarily infecting their family members) and, therefore, their vaccination is more beneficial.
- Patient protection
  - Patients are at increased risk of being infected by transmission from infected HCWs. HCW vaccination reduces this risk.
  - Vaccinating HCWs increases vaccination rates among their patients.
  - Vaccination reduces HCWs sick leave during the flu season when there is increased patient need.

The discussion of these arguments is based on critical appraisal of the few published studies, mainly randomly controlled trials, examining the effect of vaccination and on relevant systematic reviews. A Medline search was performed for studies published from 1980 to date of submission using combinations of search terms depending on the specific issue being examined, with the major terms being influenza vaccine, vaccination, immunization, health personnel, healthcare worker, absenteeism, and sick leave. The search was all inclusive with no limitation put on language or research method. Citation lists for identified studies and cited papers, including those from articles presenting evidence as to vaccination effectiveness, were also investigated, as was The Cochrane Database of Systematic Reviews. The central effort was directed at taking a fresh critical look at the trials examining patient benefit. The studies' internal validity (bias, confounding, chance effects), the correlation between their content and conclusions, and their applicability were examined.

## 3. Results and Discussion

### 3.1. Assertion: “All Adults (HCWs Included) Should Be Vaccinated against Influenza”

Commonly quoted figures demonstrating the effectiveness of influenza vaccination in healthy nonelderly adults are those from a study on randomly assigned volunteers from the Minneapolis area [6]. The rates of upper respiratory infections and of sick leave were, respectively, 25% and 36% lower among vaccinated adults compared to placebo recipients. However, the results of most other studies on the effectiveness of healthy adult vaccination are not as impressive. The 2010 Cochrane review on vaccines for preventing influenza in healthy adults [7] detected a statistically significant reduction in confirmed influenza cases, the size of which depended on the degree of vaccine matching to the circulating virus. However, the reviewers point out that the small overall average absolute difference of about 1% suggests that 100 adults would need to be vaccinated to prevent one case of influenza. The review showed that vaccine reduced time off work by an average of 0.13 days. This small effect was of borderline statistical significance (95% CI 0.00–0.25). Vaccination did not have a statistically significant effect on hospitalization or complications, and no evidence was found that vaccines prevent viral transmission. As the review included industry funded trials, the authors found it necessary to include a warning as to the interpretation of its content, stressing that the association between industry funding and study conclusions and publication, as demonstrated in a systematic review of studies on the effect of influenza vaccines [8], could have biased their results and that reliable evidence on influenza vaccines is thin but there is evidence of widespread manipulation of conclusions and spurious notoriety of the studies. The reviewers suggest that although serious harm from vaccination may be rare it cannot be ignored and conclude that the results of their literature review discourage the utilization of vaccination against influenza in healthy adults as a routine measure.

### 3.2. Assertion: “HCWs Are at Increased Risk of Infection (and Thus Also of Secondarily Infecting Their Family Members) and, Therefore, Their Vaccination Is More Beneficial” 

No reliable date could be found on influenza rates in HCWs (or their families) or comparisons to the general population [9, 10]. 

A small number of hospital based trials examined the effect of influenza vaccination on HCWs. Weingarten et al. [11], in a season with partial matching between vaccination and outbreak strains, failed to find a significant reduction of respiratory disease or sick leave among vaccinated HCWs. Wilde et al. [12] demonstrated high effectiveness of vaccinating hospital employees in preventing serologically defined influenza infection with 13.4% of control subjects and only 1.7% of vaccine recipients developing serologic evidence of influenza. However, this did not translate into clear clinical benefit—the small mean reductions in febrile respiratory disease (0.12 days) and absence from work (0.11 days) were far from reaching statistical significance. Saxén and Virtanen [13], in pediatric hospitals, also found no reduction in respiratory disease but demonstrated a statistically significant reduction of 0.4 days of sick leave because of respiratory infection in vaccinated personnel. There was no difference in absenteeism between HCWs with or without close patient contact. It should be noted that no explanation was given as to why this datum, favoring vaccination, was presented but no data were presented as to the total, all cause, absenteeism. 

In a matched cluster-randomized trial of the effect of vaccinating nursing home staff in the Paris area [14], 8.7% of HCWs reported at least one day of sick leave during the influenza season compared to 13.3% in the control arm (*P* = 0.03). It should be noted that this, and the other cluster randomized studies which are discussed later in this paper, were not blinded. Therefore, any suggested benefit in the vaccinated group was not necessarily the effect of vaccination itself but, rather, could be that of the vaccination campaign increasing awareness of influenza and leading to the taking of other preventive measures. Data supporting this argument is presented in Section 3.3. 

The small amount of data on HCW vaccination does not, therefore, support the notion that it is more effective for self-protection in this group than in the general population. No studies appear to have been performed in the setting of primary care clinics where the contact with patients is less intense. The prevailing argument that HCWs are at higher risk of infection because of their proximity to infected patients is presently theoretical. A contrary, also logical and unproven, argument could be that HCWs are more aware of the danger and take more precautions against infection, such as keeping a distance from others, hand washing, and room ventilation.

### 3.3. Assertion: “Patients Are at Increased Risk of Being Infected by Transmission from Infected HCWs. HCW Vaccination Reduces This Risk”

The most compelling argument presented for HCW vaccination is that infection of HCW puts their patients at risk of infection and therefor HCWs are morally obliged to get vaccinated. 

A number of reports have documented influenza outbreaks in hospitals [15] and nursing homes [16], showing that personnel infection preceded patient infection. This has been interpreted as proof that HCWs were the source of patient infection. However, no studies have shown that this sort of temporal relationship between staff and patient infection is more frequent than expected by chance. Furthermore, even if we were to accept that in some situations HCW infection precedes inpatient outbreaks, this is far from proving causality. The fact that infection takes longer to reach patients in a relatively closed environment is not surprising but does not show that the main vectors were HCWs (except in an isolated intensive care unit with no other contacts and no introduction of new patients), or that an infection from other sources, such as visitors or new patients, would have spread less extensively.

The heavier proof for patient benefit from HCW vaccination is considered to come from the four randomly controlled trials on elderly residents in long term care institutions [14, 17–19], comparing control homes with homes where vaccination campaigns were directed at the staff. All four studies concluded that HCW vaccination leads to a reduction in patient mortality. Because of the importance of this conclusion, these articles require special scrutiny.

The first published article [17], on geriatric long-term care hospitals in Scotland, demonstrated that staff-vaccinated hospitals had a significantly lower rate of inmate mortality (10% compared to 17% in control hospitals, OR 0.56, 95% CI 0.40–0.80) and influenza like disease. However, special inspection of the article reveals that mortality and morbidity data used for the comparison started at the end of October although the first outbreak of influenza occurred in January, over two months later. Examination of the patient mortality curves for vaccinated and unvaccinated staff shows that they diverge from the beginning of data collection, two months before the first influenza outbreak, and continue to diverge at the same rate when influenza breaks out. It is unclear why data preceding the outbreaks were included in the analysis, and the early mortality divergence clearly suggests that the difference between the two groups was unrelated to influenza. The difference could be the effect of intervention on increased awareness of the dangers of influenza, this resulting in other behavioral preventive measures which were effective also against other respiratory viruses. Further problems in the article include data inconsistencies such as a larger number of patients dying of pneumonia than the number that developed lower respiratory tract infection, the possible bias in identifying influenza-like disease by nonblinded nurses, and the fact that no difference was identified in serologic proven influenza. The article's conclusion and heading stating that “influenza vaccination of health care workers in long-term-care hospitals reduces the mortality of elderly patients” is, therefore, not supported by its content.

The second, similar, study [18] was performed two years later by the same investigating team (with some changes) in the same geographic area. Mortality during winter was 13.6% in staff-vaccinated long-term hospitals and 22.4% in control hospitals (OR 0.58, 95% CI 0.40–0.84). This is interpreted as indicating that staff vaccination substantially decreased mortality among patients. No data is presented on dates of flu outbreaks, so that the temporal relationship between influenza and mortality cannot be examined here. However, the data reveals that vaccine hospitals had both a much higher rate of vaccinated patients (48% compared to 33%) and less patient disability at baseline. Clearly, both these factors could have contributed to the lower mortality in these hospitals. A regression analysis showed that when the confounding effects of disability and patient vaccination were even partially controlled for (by using uniform average disability and vaccination scores for all residents of each hospital), the association between staff vaccination and patient mortality lost its statistical significance. Regretfully, the result of this multivariate analysis is ignored in the authors' conclusion, based on the crude data, that vaccination of HCWs is associated with a substantial decrease in patient mortality. This study, like its predecessor, did not find an association between HCW vaccination and virological proof of patient infection, despite a good match between the vaccine and outbreak influenza variants.

The third study [19] was performed in pair matched English care homes during two consecutive years. Significantly lower resident mortality (11.2% versus 15.3%, *P* = 0.002) was observed in the intervention homes in the first year but not in the second, in which influenza activity was low. Lower rates were found in the first year also for influenza like illness and admissions with influenza-like illness. However, comparison of resident characteristics demonstrates that intervention homes in that year, but not the other, had appreciably higher rates of resident vaccination (78.2% compared to 71.4%) and lower rates of highly dependent residents (36.0% and 41.4%). As in the previous study [16] this could explain much of the difference in mortality and morbidity. The article failed to examine the effect of these factors, or, as previously explained, to distinguish between the effect of vaccination and the general effect of the intervention increasing staff awareness for preventive behavior. No serologic evidence was presented to support the claim that the differences in outcomes were related to influenza. Also, disease data could have been biased by being collected by nonblinded nurses. 

The last of the four RCTs, performed on pair-matched nursing homes in Paris [14], did not show a significant difference in crude resident mortality data (5.2% in intervention and 6.0% in control homes, *P* = 0.08). However a multivariate analysis model, which included a disability score and patient vaccination status, showed that belonging to the vaccination arm was a significant predictor of resident mortality (OR 0.80, *P* = 0.02). The vaccination homes did not show lower hospitalization rates, but influenza like illness was also significantly lower. The authors point out that examining the weekly mortality rates and influenza like illness they were surprised to find that the difference between intervention and control rates was larger for a preceding RSV (respiratory syncytial virus) outbreak than for the influenza outbreak. They explain, correctly, that reduced RSV morbidity in the intervention group could not be an effect of influenza vaccination, but rather that the intervention may have made the staff in the vaccinated homes more aware of the risks of influenza, leading them to adopt general preventive measures effective also against other respiratory viruses (such as RSV). If that is the case, then why are these general effects of the intervention not accepted also as the reason for the smaller reduction in morbidity and mortality during the influenza outbreak? Examining the chart of the weekly rates in the article's supporting information shows the even more surprising information (not mentioned in the text) that most of the reduced morbidity and mortality in the study period was during the two weeks before the influenza outbreak. This difference, before the appearance of influenza, is shortly after the RSV outbreak peak and most probably related to it. Looking at the graphs, it appears that removing these two weeks, the inclusion of which remains unexplained, would cancel any substantial difference in mortality. 

To summarize these four RCTs, the repeated conclusion that staff vaccination has preventive value for elderly patients in nursing homes appears to be the result of major methodological errors and wishful thinking. Even when there appears to be less morbidity and mortality in the intervention hospitals this probably resulted from other factors. 

The severely biased conclusions of these articles are the crux of the “proof” presented by authorities supporting HCW vaccination. It is somewhat depressing to see the prejudiced manner in which the literature can be presented, as illustrated by the 2010 CDC advisory committee on immunization practices [20] recommendations on HCW vaccination. The above reviewed flawed studies are presented by this committee as evidence and further support is added by stating: “a review concluded that vaccination of HCP in settings in which patients also were vaccinated provided significant reductions in deaths among elderly patients from all causes and deaths from pneumonia.” This statement does not correctly represent the referenced 2006 review [21] which presented the flawed data from the two studies published at that time [17, 18] but actually concluded, very differently, that “…an incremental benefit of vaccinating health-care workers for elderly people has yet to be proven in well-controlled clinical trials”. This review was updated in a 2010 Cochrane systematic review [9] based on all four RCTs, which concluded that “no effect was shown for specific outcomes: laboratory proven influenza, pneumonia, and death from pneumonia. An effect was shown for nonspecific outcomes of ILI (influenza like disease), GP consultation for ILI, and all-cause mortality. These nonspecific outcomes are difficult to interpret because ILI includes many pathogens, and influenza contributes <10% of all-cause mortality in individuals >60…The identified studies are at high risk of bias…*We conclude there is no evidence that vaccinating HCWsprevents influenza in elderly patients in long term care facilities*.” This important and unambiguous conclusion was disregarded by the CDC committee in their recommendations, published six months later, favoring HCW vaccination [20].

### 3.4. Assertion: “Vaccinating HCWs Increases Vaccination Rates among Their Patients”

Studies have shown the importance of a physician's recommendation for patient vaccination [22, 23] and an association between physicians being immunized and their reported recommendations to their patient [24, 25]. Patients have more confidence in counseling from physicians who themselves demonstrate healthy behavior [26, 27] and physicians who can report that they themselves got immunized may be more successful in convincing reluctant patients. However, only a weak association was demonstrated between actual patient vaccination and their primary care physician's personal vaccination status (OR 1.08, 95% CI 1.02–1.14) in one cross-sectional study [28] and none in another [29]. 

The previously described RCTs on the effect of staff vaccination on patients in long term institutions did not specifically examine the effect of HCW vaccination on patient vaccination. Comparison of the crude patient vaccination data for intervention and control homes gives inconsistent results; only in one trial [18] was patient vaccination rate clearly higher in the institutions where the staff was vaccinated. Even if one were to suggest that the increased patient vaccination rate resulted from the intervention among the staff, this, as the authors themselves correctly imply, did not necessarily result from HCW vaccination but rather from the intervention raising HCWs' awareness of influenza risk in their patients. The vaccination rates in the other trials are even less supportive of a positive effect of staff vaccination: in one trial [17] the rate in the homes where the workers were vaccinated was significantly lower than in the controls, in one [14] it was similar and in another [19] it was significantly higher in only one of two years.

A controlled trial in primary care [30] failed to demonstrate a substantial association between raising staff vaccination rates and patient vaccination rates. 

Staff vaccination appears, therefore, not to be an important factor in increasing patient vaccination.

### 3.5. Assertion: “Vaccination Reduces HCWs Sick Leave during the Flu Season When There Is Special Patient Need”

This issue was addressed in the section on HCW flu risk, showing that vaccination leads to only a small, if any, reduction in HCW sick leave. This cannot be considered to be a proven significant benefit to patients. 

### 3.6. Is Benefit from Vaccination Uniform to All HCWs?

All four RCTs supposedly showing that staff vaccination reduces patient mortality were performed in long-term nursing institutions and, therefore, even if their conclusions were valid, they would not necessarily apply to all healthcare situations. Encounters in most other situations, for example in community clinics, are of less proximity and duration and the patients are generally healthier and at lower risk. Preventive measures which may be valuable in intensive care units or geriatric nursing homes may have no significance in healthier settings such as preventive services for healthy populations or primary care clinics. People using these services are generally mobile and in repeated and close contact with others, including family, friends, at the supermarket, in the mall, at the post office, on the bus, in waiting rooms, and at the theatre. There is no evident basis to the belief that a short encounter with community clinic HCWs substantially increases the risk of contracting influenza. The importance of vaccination may also differ according to the HCW's specific activity; vaccination may be necessary for a nurse in a hospice but superfluous for a clerk in an ambulatory dermatology clinic. HCWs, like others, with chronic disease may have greater personal benefit from vaccination.

## 4. Conclusion

The present paper examined each of the arguments in favor of HCW influenza vaccination and showed that they are not supported by existing literature. The evidence base supporting vaccination is unsound and prejudiced.

The personal benefit from vaccinating healthy nonelderly adults is small and there is no evidence to show that it is any different for HCWs. The studies aiming to prove the widespread belief that staff vaccination has a substantial effect on patient morbidity and mortality are heavily flawed. No reliable evidence shows that HCW vaccination has noteworthy advantage to their patients—not in reducing patient morbidity or mortality, not in increasing patient vaccination, and not in decreasing HCW work absenteeism.

The finding that there is no valid evidence clearly supporting vaccination of HCWs does not mean that there cannot be some unproven benefit from vaccination. However, if substantial benefit exists it still needs to be demonstrated in valid studies.

This paper is of special importance due to the increasing pressure to mandate HCW vaccination. Such drastic action, at the expense of personal freedom, should not be accepted in the absence of very strong evidence for a very strong population benefit. The decision whether to vaccinate is, at present and in most situations, not a moral issue and should remain that of the individual HCW, preferably based on real information.

## Key Points

- There are no studies showing that healthcare workers are at increased risk of influenza and its complications or that the vaccine is more effective in this group.
- The evidence base for the claim that vaccinating healthcare workers against influenza protects their patients is heavily flawed and inconclusive at best.
- The benefit from vaccinating healthcare workers, if any, may differ according to specifics of the patients, location, and worker.
- At present, the decision whether to get vaccinated should, except possibly in extreme situations, be that of the individual healthcare worker, without legal, institutional, or peer coercion.
